# The translatome of neuronal cell bodies, dendrites, and axons

**DOI:** 10.1073/pnas.2113929118

**Published:** 2021-10-20

**Authors:** Caspar Glock, Anne Biever, Georgi Tushev, Belquis Nassim-Assir, Allison Kao, Ina Bartnik, Susanne tom Dieck, Erin M. Schuman

**Affiliations:** ^a^Department of Synaptic Plasticity, Max Planck Institut fur Hirnforschung, Frankfurt am Main, Hessen 60438, Germany

**Keywords:** translatome, local protein synthesis, dendrites, RNA localization

## Abstract

To form synaptic connections and store information, neurons continuously remodel their proteomes. The impressive length of dendrites and axons imposes logistical challenges to maintain synaptic proteins at locations remote from the transcription source (the nucleus). The discovery of thousands of messenger RNAs (mRNAs) near synapses suggested that neurons overcome distance and gain autonomy by producing proteins locally. It is not generally known, however, if, how, and when localized mRNAs are translated into protein. To investigate the translational landscape in neuronal subregions, we performed simultaneous RNA sequencing (RNA-seq) and ribosome sequencing (Ribo-seq) from microdissected rodent brain slices to identify and quantify the transcriptome and translatome in cell bodies (somata) as well as dendrites and axons (neuropil). Thousands of transcripts were differentially translated between somatic and synaptic regions, with many scaffold and signaling molecules displaying increased translation levels in the neuropil. Most translational changes between compartments could be accounted for by differences in RNA abundance. Pervasive translational regulation was observed in both somata and neuropil influenced by specific mRNA features (e.g., untranslated region [UTR] length, RNA-binding protein [RBP] motifs, and upstream open reading frames [uORFs]). For over 800 mRNAs, the dominant source of translation was the neuropil. We constructed a searchable and interactive database for exploring mRNA transcripts and their translation levels in the somata and neuropil [MPI Brain Research, The mRNA translation landscape in the synaptic neuropil. https://public.brain.mpg.de/dashapps/localseq/. Accessed 5 October 2021]. Overall, our findings emphasize the substantial contribution of local translation to maintaining synaptic protein levels and indicate that on-site translational control is an important mechanism to control synaptic strength.

## Significance

Proteins are the key drivers of neuronal synaptic function. The regulation of gene expression is important for the formation and modification of synapses throughout the lifespan. The complexity of dendrites and axons imposes unique challenges for protein supply at remote locations. The discovery of messenger RNAs (mRNAs) and ribosomes near synapses has shown that local protein synthesis represents an important solution to this challenge. Here we used RNA sequencing and ribosome sequencing to determine directly the population of mRNAs that is present and in the process of translation in neuronal cell bodies, dendrites, and axons. Thousands of transcripts were differentially translated between the cell body and synaptic regions with over 800 mRNAs exhibiting more translation in the dendritic–axonal compartment.

At neuronal synapses, more than 2,500 proteins (1, 2) (the “synaptic proteome”) act as sensors and effectors to control neuronal excitability, synaptic strength, and plasticity. The elaborate morphology and functional compartmentalization of the individual neuron imposes unique logistical challenges to maintain and modify the synaptic proteome at locations remote from the transcription source (i.e., the nucleus). To fulfill the local demand for new protein, neurons localize messenger RNAs (mRNAs) and ribosomes near synapses to produce proteins directly where they are needed (1). Using high-throughput sequencing, several groups have reported the localization of thousands of transcripts to axons and dendrites (the “local transcriptome”) (3–7). In many cell types, however, it has been shown that the transcript levels do not always predict protein levels (8), suggesting that mRNA translation is a highly regulated process. Since proteins, rather than mRNAs, drive cellular function, it is imperative to determine directly which transcripts are translated into proteins in dendrites and/or axons in vivo (the “local translatome”). Importantly, it remains unknown which transcripts exhibit differential levels of translation between somatic and synaptic regions.

A given transcript’s translation level is determined by the rate of ribosome recruitment to the start codon during initiation and the velocity of ribosome translocation during polypeptide elongation. For most mRNAs, translation initiation is considered rate limiting (9): Initiation is regulated by elements within the mRNA’s untranslated regions (UTRs) that bind RNA-binding proteins (RBPs) or miRNAs (10–12). In addition, the elongation rate also plays a regulatory role in determining the amount of protein produced from a transcript (13). Although disrupted translational control has been linked to a number of neurological disorders (14), little is known about the magnitude and mechanisms for transcript-specific translational regulation in neuronal compartments.

In this study, we combined deep sequencing of ribosome-protected fragments (ribosome sequencing [Ribo-seq]) and RNA sequencing (RNA-seq) of microdissected hippocampal rodent brain sections to provide a comprehensive analysis of the mRNA translational landscape both in the somata (enriched in cell bodies) and the neuropil (enriched in neuronal dendrites/axons). Thousands of mRNAs were translated in the somatic and synaptic regions. Many transcripts exhibited differential translation levels between somatic and synaptic regions. Many of these translational changes likely resulted from differences in the RNA levels between the somata and neuropil. Furthermore, we found evidence for pervasive translational regulation of synaptic proteins in both neuronal compartments. We provide a dynamic query-based web interface for exploring mRNA transcripts and their translation in neuronal compartments (15). Together, our results reveal an unprecedented capacity for local protein production in vivo to maintain and modify the pre- and postsynaptic proteome.

## Results

### Measuring Transcriptome-Wide Translation in Neuronal Compartments.

**Fig. 1.**
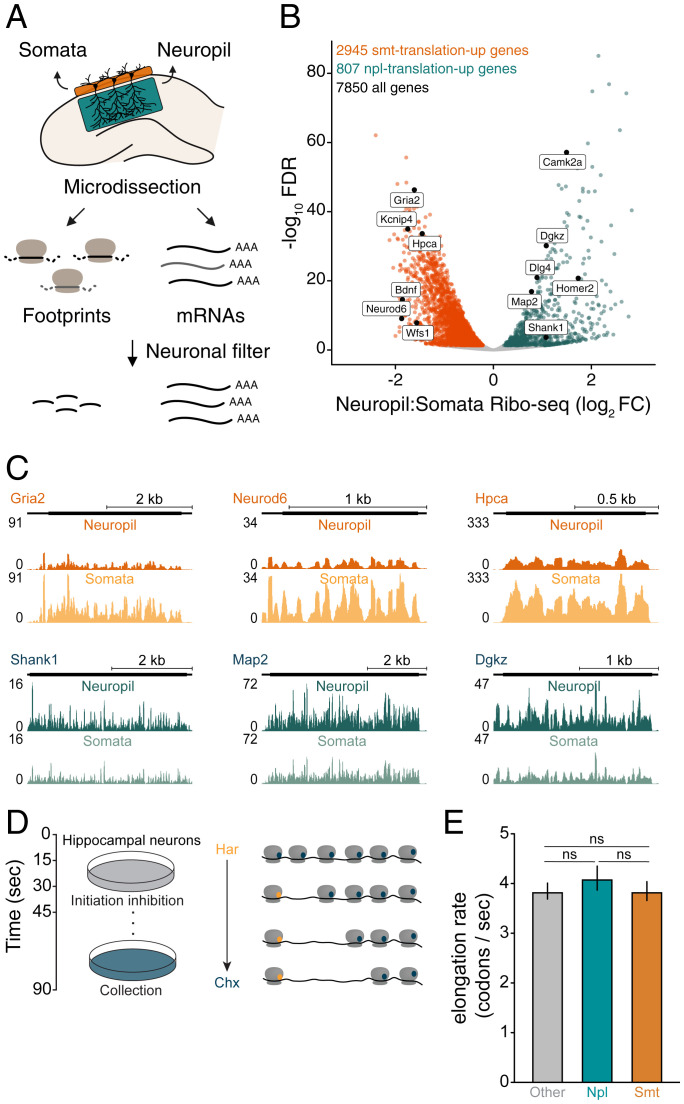
Many transcripts display differential translation between the somata and neuropil. (*A*) Experimental workflow. Microdissection of the CA1 region of the rat hippocampus. RNA-seq and Ribo-seq were conducted simultaneously for the somata (enriched in pyramidal neuron cell bodies) and the neuropil (enriched in dendrites and axons) layers. A neuronal filter was applied to enrich for excitatory neuron transcripts in downstream analyses. (*B*) Volcano plot comparing the translational level of 7,850 transcripts between compartments (neuropil:somata Ribo-seq ratio [log_2_FC]). FDR < 0.05 using DESeq2 (*Experimental Procedures*). Colored dots highlight the transcripts significantly more translated in the somata (somata [smt]-translation-up, *n* = 2,945, orange) or neuropil (neuropil [npl]-translation-up, *n* = 807, teal). (*C*) Coverage tracks representing the average neuropil (*Top*) or somata (*Bottom*) ribosome footprint coverage for candidate smt-translation-up (*Gria2*, *Neurod6*, and *Hpca*) and npl-translation-up (*Shank1*, *Map2*, and *Dgkz*) transcripts. The *y* axis indicates the number of normalized reads. (*D*) Schematic depicting in vivo ribosome run-off following harringtonine incubation of rat hippocampal cultures. (*E*) Elongation rates for smt-translation-up (orange), npl-translation-up (teal), and other (gray) transcripts inferred from the slope of the linear fit shown in *SI Appendix*, Fig. S4 are plotted with their SE (*n* = 3). *P* = 0.5738, One-way ANOVA. Har, harringtonine; Chx, cycloheximide; ns, not significant.

To discover the mRNA species localized and translated in cell bodies as well as dendrites and axons we carried out a genome-wide analysis of the transcriptome and translatome of the somata and neuropil from microdissected hippocampal slices (16). Ribosome footprints were obtained from somata and neuropil lysates to assess the number and position of translating ribosomes on a transcript (Ribo-seq) (17). In parallel, transcript levels were quantified by performing RNA-seq from the somata and neuropil (Fig. 1*A*) (16). The RNA- and Ribo-seq libraries from the somata and neuropil were highly reproducible among the three biological replicates (*SI Appendix*, Fig. S1 *A* and *B*). Furthermore, the Ribo-seq samples exhibited the expected depletion of footprint read densities in the UTRs and introns of transcripts (*SI Appendix*, Fig. S1 *C* and *D*), as well as three-nucleotide phasing (*SI Appendix*, Fig. S1 *E* and *F*) (17).

We detected 13,055 and 12,371 transcripts with one count per million (CPM) in two of three neuropil (*SI Appendix*, Fig. S2*A*) or somata (*SI Appendix*, Fig. S2*B*) Ribo-seq replicates, respectively. Using the Ribo-seq datasets, we found substantial overlap between our translatome data and a previously published neuropil (*SI Appendix*, Fig. S2*A*) and somata (*SI Appendix*, Fig. S2*B*) transcriptome (3). The somata and neuropil of the hippocampus contain excitatory neuron cell bodies and their processes, as well as glia and interneurons. We created a pipeline to focus on excitatory neuron genes by minimizing the contribution of other cell types via bioinformatic filtering. To obtain a comprehensive set of glia-enriched transcripts, we prepared hippocampal neuron- and glia-enriched cultures (*SI Appendix*, Fig. S2*C* and Dataset S1). Because the somata and neuropil do not only contain glia but also interneurons, we additionally compiled lists of transcripts enriched in nonexcitatory neuron cell types in the hippocampus. To do so, we identified the transcripts significantly deenriched in the hippocampi of two different RiboTag mouse lines that target primarily excitatory neurons: Camk2Cre::RiboTag mice (*SI Appendix*, Fig. S2*D*), as well as the microdissected somata (*SI Appendix*, Fig. S2*E*) and neuropil (*SI Appendix*, Fig. S2*F*) from Wfs1Cre::RiboTag mice (16). Combining these datasets, we obtained a list of “contaminant” nonexcitatory neuron genes (*SI Appendix*, Fig. S2*G*).

### Many Transcripts Exhibit Differential Translation between Neuronal Compartments.

The number of ribosomes loaded on a transcript indicates how much it is translated. To identify transcripts that exhibit differential translation between the somata and neuropil, we computed neuropil:somata Ribo-seq ratios (DESeq2) (18) (*Experimental Procedures*). After subtraction of the contaminant genes, we detected 7,850 neuronal transcripts (*SI Appendix*, Fig. S2*H*) (19) that were translated in both the somata and neuropil (Fig. 1*B*). Of these, 807 transcripts exhibited significantly increased translation levels in the neuropil compared to the somata (“neuropil-translation-up”) (Fig. 1*B* and Dataset S2). The neuropil-translation-up transcripts included, for example, *Shank1*, *Map2*, and *Dgkz* (Fig. 1 *B* and *C*). In contrast, 2,945 transcripts showed increased translation in the somata, including *Gria2*, *Neurod6*, and *Hpca* (“somata-translation-up”) (Fig. 1 *B* and *C* and Dataset S2). Both neuropil- and somata-translation-up transcripts exhibited three-nucleotide periodicity arising from the codon-by-codon translocation of ribosomes along mRNAs during translation in the neuropil and somata, respectively (*SI Appendix*, Fig. S3 *A* and *B*). Consistent with previous findings (12), the neuropil-translation-up transcripts displayed significantly longer 3′ UTRs (*SI Appendix*, Fig. S3*C*).

Previous studies suggested that mRNAs present in dendrites and/or axons might be translationally silenced, via the “pausing” of ribosomes at the level of elongation (13, 20). To address this, we asked whether the neuropil- and somata-translation-up transcripts exhibited differences in the speed of translation elongation. We performed a time series of ribosome “run-off” by incubating cultured hippocampal neurons for 15, 30, 45, or 90 s with harringtonine, a drug that immobilizes ribosomes immediately after translation initiation, resulting in a progressive run-off of ribosomes over time (Fig. 1*D* and *SI Appendix*, Fig. S4). We analyzed the rate of ribosome progression (elongation) from the 5′ end of neuropil- and somata-translation-up transcripts (*SI Appendix*, Fig. S4). The neuropil- and somata-translation-up transcript subsets displayed a similar elongation rate of ∼4 codons per second (Fig. 1*E* and *SI Appendix*, Fig. S4), a value that is within the range measured in other cell types (3 to 10 codons per second) (21–24). Together, these findings indicate that neuropil-translation-up mRNAs are globally not significantly more paused than other transcripts.

**Fig. 2.**
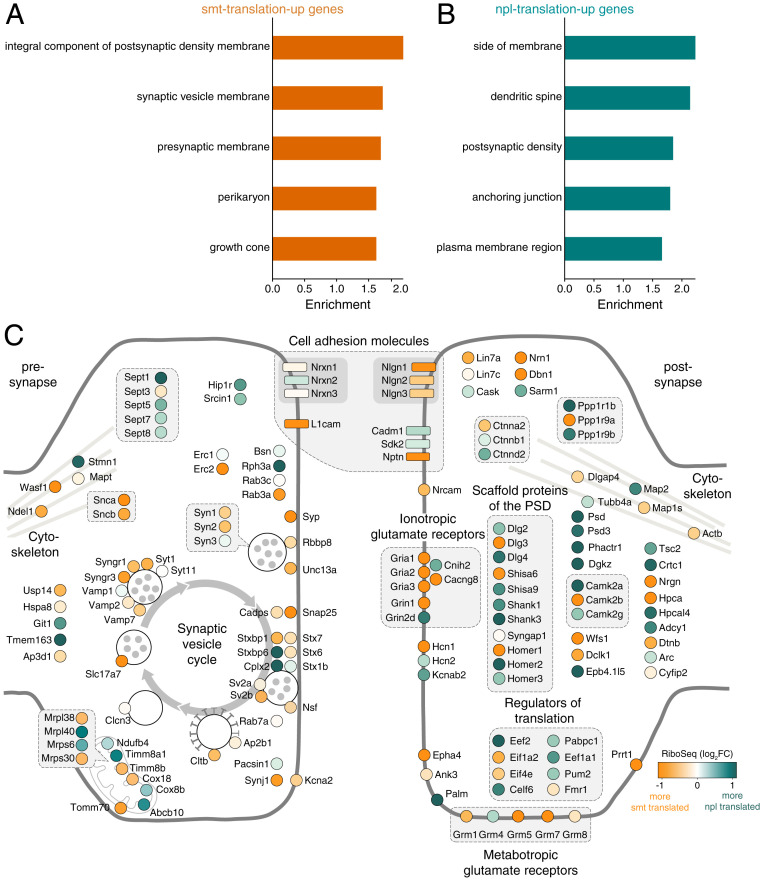
Functional segregation of transcripts differentially translated between the somata and neuropil. (*A* and *B*) GO terms representing the top five highest significantly enriched (FDR < 0.05) protein function groups for somata-translation-up (*A*) and neuropil-translation-up (*B*) transcripts. (*C*) Scheme depicting proteins of glutamatergic synapses. Ribo-seq neuropil:somata ratios (log_2_FC) are color coded from orange (more somata-translated) to teal (more neuropil-translated). Interacting proteins are displayed in closer proximity. Proteins with similar functions are grouped together and the synaptic vesicle cycle is indicated by arrows.

To examine whether particular protein function groups are encoded by transcripts that exhibit increased translation levels in either compartment, we performed a gene ontology (GO) analysis (Fig. 2 *A* and *B*). An enrichment of terms associated with synaptic function was found for both somata- and neuropil-translation-up transcripts (Fig. 2 *A* and *B*). For the somata-translation-up transcripts, we observed a significant overrepresentation of the term “perikaryon” as well as many membrane-related terms such as “integral component of postsynaptic density membrane,” “presynaptic membrane,” or “synaptic vesicle membrane” (Fig. 2*A*). On the other hand, mostly postsynaptic functions were significantly associated with the neuropil-translation-up transcripts, including for example “dendritic spine” and “postsynaptic density” (Fig. 2*B*). To understand better the synaptic function of the neuropil- and somata-translation-up transcripts, we analyzed the neuropil:somata Ribo-seq fold changes of excitatory synaptic proteins (Fig. 2*C*). We noted that ionotropic and metabotropic glutamate receptor subunits (AMPARs, NMDARs, and mGluRs) mostly displayed greater translation levels in the somata (Fig. 2*C*). In contrast, many glutamate receptor-associated accessory (e.g., *Cnih2*) or scaffold proteins (e.g., *Shank1*,* Dlg4*, and *Homer2*) exhibited increased translation levels in the neuropil (Fig. 2*C*). Also, we found that many presynaptic proteins exhibited greater protein synthesis rates in the somata (Fig. 2*C*). Interestingly, we identified several nuclear-encoded mRNAs related to mitochondrial function that exhibited enhanced translation levels in the neuropil (e.g., *Timm8a1* and *Mrpl40*) (Fig. 2*C*).

The mRNA transcript and translation profiles in the somata and neuropil are available for download and exploration at a searchable web interface (https://public.brain.mpg.de/dashapps/localseq/). This interactive database allows viewers to compare transcript and mRNA translation levels between neuronal compartments.

### Most Translational Changes between Somatic and Synaptic Regions Can Be Explained by Differences in RNA Abundance.

**Fig. 3.**
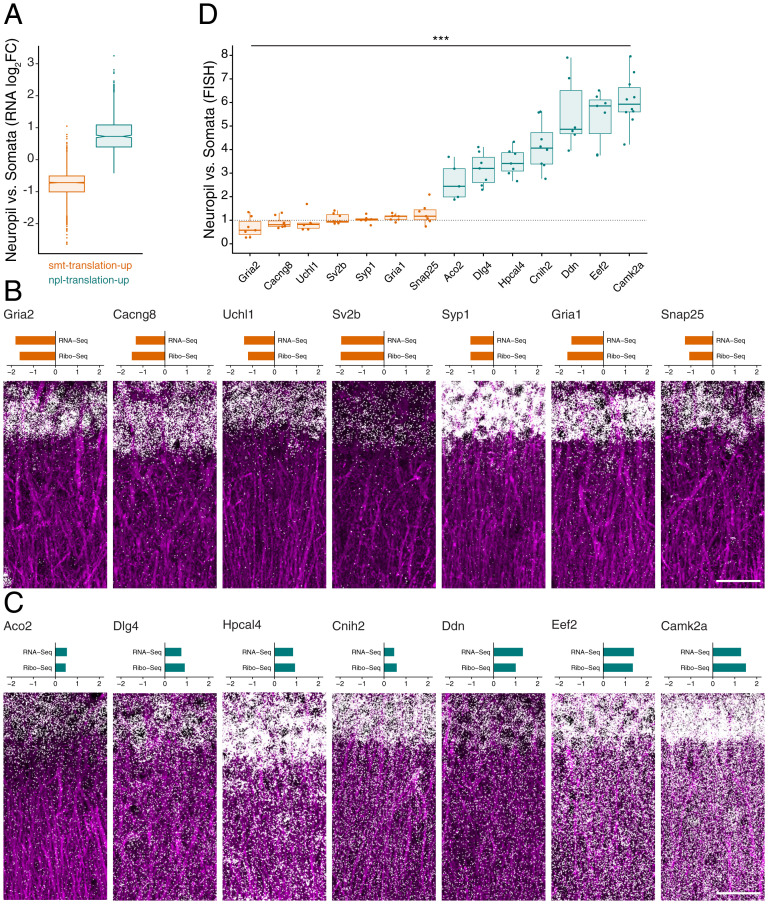
Differential translation of neuropil- and somata-translation-up genes is accompanied by between-compartment changes in RNA levels. (*A*) Box plot representing the neuropil:somata RNA-seq ratio (log_2_FC) for somata (smt)-translation-up (orange) and neuropil (npl)-translation-up (teal) genes (DESeq2; *Experimental Procedures*). (*B* and *C*) (*Top*) Neuropil:somata RNA-and Ribo-seq ratios (log_2_FC) for candidate smt-translation-up genes (*Gria2*, *Cacng8*, *Uchl1*, *Sv2b*, *Syp1*, *Gria1*, and *Snap25*) (*B*) and npl-translation-up genes (*Aco2*, *Dlg4*, *Hpcal4*, *Cnih2*, *Ddn*, *Eef2*, and *Camk2a*) (*C*). (*Bottom*) FISH signal in the CA1 region of rat hippocampal slices using probes against smt- (*B*) and npl-translation-up (*C*) candidate genes. The dendrites were immunostained with an anti-MAP2 antibody (purple). (Scale bar, 50 μm.) (*D*) Neuropil:somata ratio of mRNA puncta relative to the mean neuropil:somata ratio of the smt-translation-up genes (***P < 2.2e-16, Mann–Whitney *U* Test between all smt-translation-up and all npl-translation-up genes).

The translation level of a given transcript is proportional to its abundance and its ribosome density. We thus asked whether differential translation of somata- and neuropil-translation-up transcripts was associated with between-compartment changes in RNA levels (Dataset S3). Indeed, neuropil-translation-up transcripts displayed significantly higher neuropil:somata RNA-seq ratios compared to somata-translation-up genes (Fig. 3*A*). In order to validate these observations in situ in hippocampal slices, we performed high-resolution fluorescence in situ hybridization (FISH) for 14 candidate transcripts with significantly different translation levels between the somata and neuropil (Fig. 3 *B*–*D*). The in situ hybridization signal detected was highest in expected compartment (i.e., somata for somata-translation-up, Fig. 3 *B* and *D*, and neuropil for neuropil-translation-up, Fig. 3 *C* and *D*). Taken together, both the RNA-seq and FISH analyses revealed that increased translation in the somata or neuropil was accompanied by higher RNA levels in the same neuronal compartment.

**Fig. 4.**
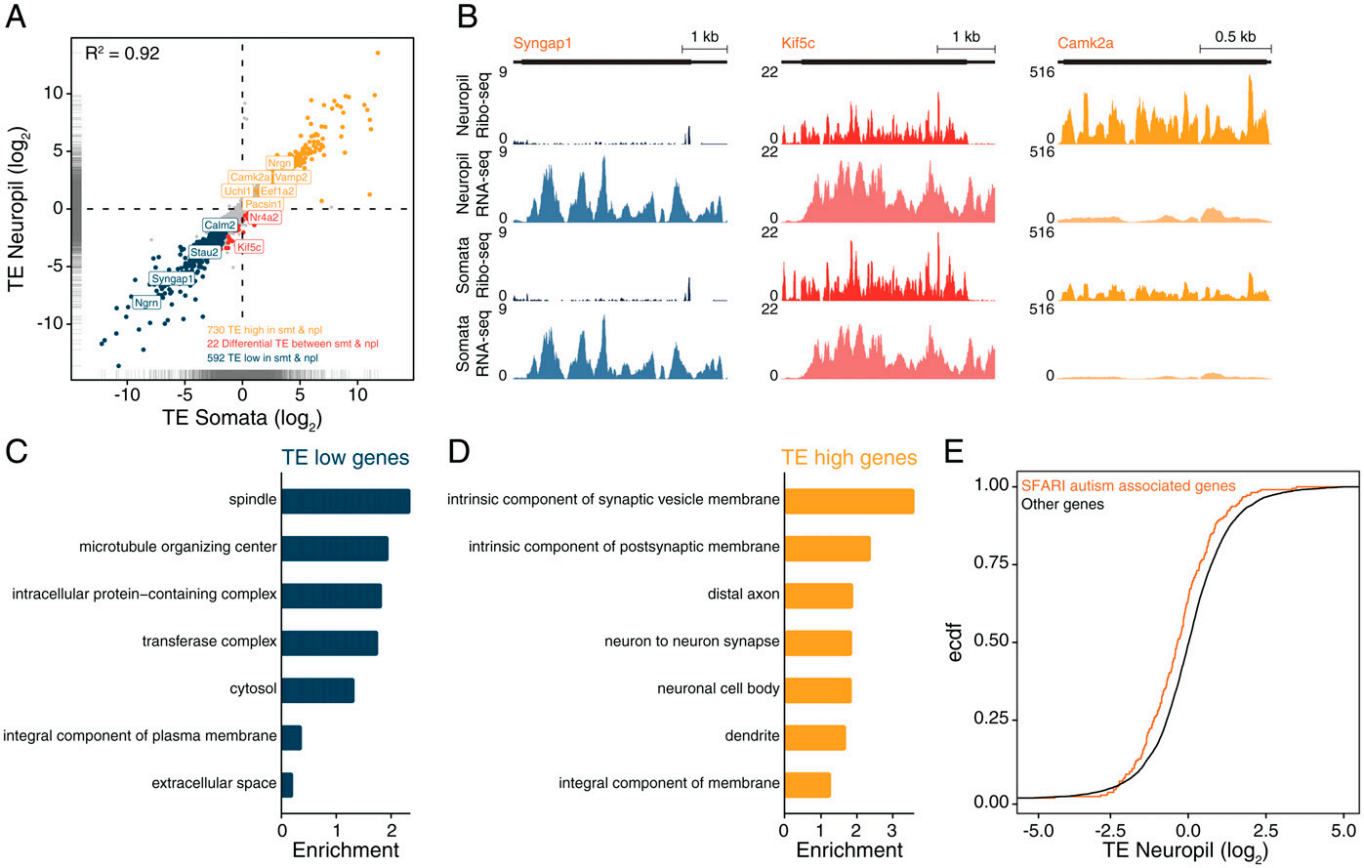
Most transcripts exhibit similar translational efficiency in the somata and neuropil. (*A*) Correlation of the translational efficiencies (TE; log_2_Ribo-Seq/RNA-seq) in the neuropil and somata (*R*^2^ = 0.92, *P* < 2.2e-16). Highlighted are genes with significantly higher (TE_high_, yellow) or lower (TE_low_, blue) TE than log_2_ 1.5 (FDR < 0.05, DESeq2) in both somata and neuropil. Genes with significantly differential TE between somata and neuropil are shown in red. DESeq2 with FDR <0.05. Marginal rug (gray) represents the distribution of the TE values in the somata (*x* axis) and neuropil (*y* axis). (*B*) Coverage tracks representing the average ribosome footprint or RNA coverage for candidate genes (*Syngap1*, *Kif5c*, and *Camk2a*) in the neuropil and somata. The *y* axis indicates reads per million (RPM). (*C* and *D*) GO terms representing significantly enriched (FDR < 0.05) protein function groups for TE_low_ (*C*) and TE_high_ (*D*) transcripts. (*E*) Empirical cumulative distribution frequency (Ecdf) of the TE (log_2_FC) of SFARI autism associated (yellow) and other (black) genes. *P* = 2.579e-05, Kolmogorov–Smirnov test.

We next compared gene-level translation efficiencies (TEs) between the neuropil and somata by computing the ratio of ribosome footprints (from Ribo-seq) to mRNA fragments (from RNA-seq) (17) in both compartments (Fig. 4*A* and Dataset S4). We observed a good correlation between the somata and neuropil TE values, indicating that most transcripts exhibit similar translational regulation in both neuronal compartments (Fig. 4*A*, *R*^2^ = 0.92, *P* < 2.2e-16). For instance, *Syngap1* exhibited low footprint-to-mRNA ratios in both somata and neuropil, indicating the relatively poor translational efficiency of this transcript (Fig. 4 *A* and *B*). In contrast, *Camk2a* was found translated with high efficiency (high footprint-to-mRNA ratio) in both neuronal compartments (Fig. 4 *A* and *B*). We also identified a handful of mRNAs that displayed significantly higher TE values in the somata, including, for example, *Kif5c* (Fig. 4 *A* and *B*). Thus, many but not all of the between-compartment differences in ribosome footprint levels can be accounted for by differences in the amount of mRNA present.

### Pervasive Translational Regulation in the Somata and Neuropil.

**Fig. 5.**
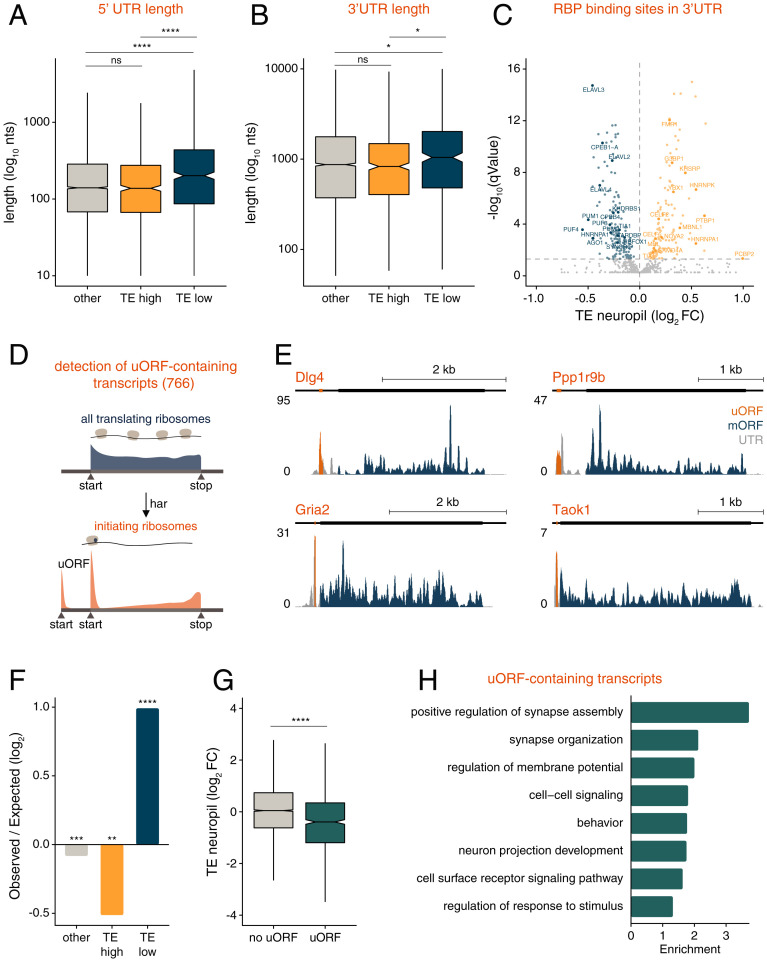
Features of translationally regulated transcripts in the somata and neuropil. (*A* and *B*) Box plots of 5′ UTR (*A*) and 3′ UTR (*B*) length (log_10_ nucleotides (nts) for TE_high_ (yellow), TE_low_ (blue), and other (gray) genes. Bars indicate 1.5*IQR. *P < 0.05, ****P < 0.0001; one-way ANOVA test followed by pairwise *t* test with Benjamini–Hochberg *P* value adjustment. (*C*) Shown are RBP motifs within 3′ UTRs associated with significantly lower (blue) or higher (yellow) neuropil TE values (q values < 0.05; Wilcoxon rank sum test) (*Experimental Procedures*). (*D*) Detection of translated uORFs in hippocampal neurons. Translation initiation sites were mapped using the drug harringtonine (har), which accumulates ribosomes at start codons. A total of 766 uORF-containing neuronal transcripts were detected in the somata and neuropil. (*E*) Coverage tracks representing the average ribosome footprint reads along the UTRs (gray), detected uORFs (orange), or the main protein coding sequence (blue) of *Dlg4*, *Gria2*, *Taok1*, and *Ppp1r9b* in the neuropil. The *y* axis indicates reads per million (RPM). (*F*) Observed-to-expected ratio of TE_high_ (teal), TE_low_ (blue), and other (gray) transcripts containing uORFs. **P < 0.01, ***P < 0.001, ****P < 0.0001; hypergeometric test. (*G*) Neuropil TE (log_2_FC) measurements of transcripts containing translated uORFs (“uORF”) or not (“no uORF”). ****P < 0.0001; Welch two-sample *t* test. (*H*) GO terms representing the top eight significantly (FDR < 0.05) enriched protein function groups for uORF-containing transcripts in the neuropil.

In both neuronal compartments, we observed a wide distribution of translation efficiencies, with a greater than 1,000-fold difference between the most and least efficiently translated transcripts in the neuropil (Fig. 4*A*). We identified 730 and 592 transcripts exhibiting significantly high or low translational efficiencies, respectively, in both somata and neuropil (Fig. 4*A* and Dataset S4). We identified gene features associated with these two groups which we call TE_low_ and TE_high_. GO analysis revealed an enrichment of terms such as “spindle” and “microtubule organizing center” for TE_low_ genes (Fig. 4*C*). In contrast, TE_high_ genes were associated with terms such as “intrinsic component of synaptic vesicle membrane” and “intrinsic component of postsynaptic membrane” (Fig. 4*D*). As a group, TE_low_ transcripts had longer coding sequences (CDS), consistent with previous observations (25–27) (*SI Appendix*, Fig. S5*A*). Because autism risk factor genes have been described to be exceptionally long (28–30), we analyzed the TE values of Simons Foundation Autism Research Initiative (SFARI) transcripts. We found that SFARI transcripts displayed overall lower TE values compared to other genes (Fig. 4*E*). The efficiency of mRNA translation is also influenced by elements within the UTRs that serve as binding platforms for regulatory RBPs (10, 12). Because longer UTRs harbor more cis-acting elements (10, 12), we examined the 5′ and 3′ UTR length of the translationally regulated transcripts. We found that TE_low_ genes exhibited significantly longer 5′ and 3′ UTRs (Fig. 5 *A* and *B*). To identify potential RBPs for the neuropil UTRs, we searched for known RBP consensus motifs (31) and determined whether transcript groups sharing the same motifs were associated with higher or lower TE values in the neuropil (*Experimental Procedures*). A total of 131 3′ UTR motifs targeted by 52 RBPs (Dataset S5) were associated with transcripts displaying significantly higher TE values in the neuropil (Fig. 5*C*; for somata see *SI Appendix*, Fig. S5*B* and Dataset S6). For example, consistent with their described role as translational enhancers (32–34), HNRNPK and MBNL1 motifs were detected in transcripts exhibiting significantly higher TE values (Fig. 5*C*). On the other hand, 155 3′ UTR motifs targeted by 90 RBPs (Dataset S5) were associated with transcripts exhibiting significantly lower neuropil TE values in the neuropil (Fig. 5*C*). Among these, we identified, for example, the CPEB, Hu (Elav), and PUF/Pumilio RBP families, all known for their repressive action on translation in neuronal processes (35). We note that none of the RBP motifs we detected within neuropil 5′ UTRs were associated with transcripts displaying significantly higher or lower neuropil or somata TE (Datasets S7 and S8). Our results thus reveal the identity of potentially novel regulators that bind the 3′ UTR and control translation, either directly or indirectly for example via the regulation of polyadenylation (34) or mRNA decay (35).

Upstream open reading frames (uORFs) also play an important role in regulating the translation of the main protein coding sequence (36). While most uORFs are believed to exert a negative effect on the translation of downstream ORFs (36), a few examples of positive-acting uORFs have been reported (37, 38). We identified translated uORFs in neuronal compartments using an integrated experimental and computational approach. To map upstream translation initiation sites within neuronal transcripts, we performed Ribo-seq on neurons treated with the drug harringtonine, which causes the accumulation of ribosomes at start codons (21) (Fig. 5*D* and *Experimental Procedures*). We then used the ORF-RATER pipeline to identify and quantify translated uORFs in the neuropil- and somata Ribo-seq data (*Experimental Procedures*) (39). In total, we identified 766 uORF-containing mRNAs in neuronal compartments (Fig. 5*D* and Dataset S9), including novel (e.g., *Gria2*, *Taok1*, *Dlg4*, and *Ppp1r9b*) (Fig. 5*E* and *SI Appendix*, Fig. S5*C*) and previously described (e.g., *Atf4* and *Ppp1r15b*) (38, 40) (*SI Appendix*, Fig. S5*D*) transcripts. A comparison of TE_low_ and TE_high_ transcripts revealed an overrepresentation of uORF-containing transcripts in the TE_low_ group and an underrepresentation of uORF-containing transcripts in the TE_high_ group (Fig. 5*F*). Additionally, uORF-containing transcripts displayed a significantly lower neuropil median TE value when compared with non-uORF-containing mRNAs (Fig. 5*G* and *SI Appendix*, Fig. S5*E* for the somata). Using the neuropil Ribo-seq data, we next computed a relative uORF to CDS ribosome density for each uORF. Of interest, the relative uORF:CDS ribosome densities ranged from 0.1 to 1,000, indicating a wide spread in the uORF-mediated translational repression in the neuropil (*SI Appendix*, Fig. S5*F*). Many uORFs displayed uORF:CDS ribosome density ratios greater than 1, indicating that uORFs often act as CDS translational repressors. A GO analysis indicated that above described uORF-containing neuropil and somata mRNAs were significantly enriched for terms like “positive regulation of synapse assembly,” “regulation of membrane potential,” and “behavior” (Fig. 5*H*). These findings highlight uORFs as an important translational regulatory element present in many transcripts in somatic and synaptic regions.

## Discussion

Using ribosome profiling, we detected thousands of mRNA species that are translated in synaptic regions, dramatically expanding the contribution of ongoing local protein synthesis to the protein pool detected in dendrites, axons, or synapses (41–44). Indeed, among the locally translated mRNAs, we identified most protein families, including signaling molecules (kinases or phosphatases), ion channels, metabotropic and ionotropic receptors, cell adhesion molecules, scaffold proteins, as well as regulators of cytoskeleton remodeling or translation.

Many transcripts were found differentially translated between neuronal compartments. An open question in the field has concerned the contribution of local synthesis to the total pool of a particular protein. Our data indicate that most proteins are synthesized in both compartments. We note that over 800 mRNAs displayed enhanced translation levels in the neuropil, suggesting that most of these proteins arise from a local source. For many transcripts, the abundance of the mRNA was positively associated with the translation level differences between somata and neuropil, as observed previously in developing neurons derived from mouse embryonic stem cells (45). Notably, the neuropil-translation-up transcripts often encoded signaling and scaffold proteins that play an important role in the maintenance and modification of synaptic strength. Of interest, we detected several mitochondrial mRNAs that displayed enhanced neuropil translation. Recently, it has been shown that endosomes can act as platforms for the local translation of candidate mitochondrial mRNAs (46). It is thus tempting to hypothesize that local translation plays a role in sustaining mitochondria, which in turn fuel protein synthesis near synapses during plasticity (47). Together, our results suggest that the increased translation levels of a specific transcript subset in the neuropil likely provide a means to ensure the efficient production of key synaptic proteins at very remote locations from the cell body.

In contrast the transcripts with increased translation levels in the somata often encoded transmembrane proteins. This protein class is typically processed through multiple membrane-bound organelles (including the endoplasmic reticulum [ER] and Golgi apparatus [GA]), where they are folded, assembled, and biochemically modified prior to their delivery to the neuronal cell surface (48). However, recent studies reported that hundreds of neuronal surface proteins (e.g., the AMPAR subunit GluA1) bypass GA maturation and likely travel directly from the ER to the neuronal cell surface (49, 50). Thus, although the bulk synthesis and posttranslational modification of transmembrane proteins might occur in the somatic ER and GA, a small residual fraction of this protein class could undergo “on demand” local translation to fine tune synaptic strength.

Using a combination of microdissection with Ribo-and RNA-seq, we found that most transcripts exhibit similar translational regulation in the somata and neuropil. In both neuronal compartments, we detected widespread translational regulation, with an unexpectedly high dynamic range in the translation efficiencies of transcripts. Among the mechanisms that regulate the synthesis of proteins in somatic and synaptic regions, we identified uORF-mediated translational control. This finding is in good agreement with previous studies revealing the role of uORFs in the translational regulation of two candidate transcripts in neuronal processes (51, 52). uORF-mediated translational control is often fine tuned by the phosphorylation of eukaryotic initiation factor 2α (eIF2α) (53). The phosphorylation of eIF2α inhibits global translation while leading to a paradoxical increase in the translation of a subset of uORF-bearing transcripts (54). Many manipulations of cellular and synaptic activity modulate the phosphorylation status of eIF2α in neurons in vivo and in vitro (54–57). Thus, activity-driven eIF2α phosphorylation could act as a switch to enhance the local translational efficiency of uORF-containing transcripts encoding key plasticity-related proteins. It is noteworthy that the translational regulation of some uORF-containing transcripts is insensitive to changes in the eIF2α phosphorylation status (e.g., the protein phosphatase 1 regulatory subunit CReP [*Ppp1r15b*]) (40).

Electron microscopy (EM) studies have shown that the distribution of the ribosomes along neuronal processes is heterogeneous, with a selective localization of protein-making machines (i.e., polyribosomes, more than three ribosomes per mRNA) beneath synapses, while only a few polyribosomes could be observed in CA1 dendritic shafts (58, 59). Dendritic shafts could be mostly populated by monosomes (i.e., single ribosome per mRNA) that cannot be visualized by EM but also represent active protein making machines in synaptic regions (16). Indeed, a recent superresolution study which likely detects both monosomes and polysomes identified a greater ribosome density in dendrites compared to EM studies (60). These observations raise intriguing questions about the definition of local translation compartments: Are different protein species synthesized within distinct subregions of neuronal processes (e.g., spines vs. dendritic shafts)? And: Could the translation efficiency of the same transcript vary depending on whether it is localized beneath synapses or in other dendritic regions? These questions set the stage for future studies characterizing the translational landscape in neuronal subregions with greater spatial resolution using, for example, proximity-specific ribosome profiling.

## Experimental Procedures

### Animals.

Timed pregnant specific-pathogen-free (Charles River Laboratories) female rats were housed in Max Planck Institute for Brain Research animal facility for 1 wk on a 12/12-h light/dark cycle with food and water ad libitum until the litter was born. Cultured neurons were derived from P0 (postnatal day 0) Sprague-Dawley rat pups (both male and female, research resource identifier: 734476). Pups were killed by decapitation. The housing and killing procedures involving animal treatment and care were conducted in conformity with the institutional guidelines that are in compliance with national and international laws and policies (Directive 2010/63/EU; German animal welfare law; Federation of European Laboratory Animal Science Associations guidelines). The animals were killed according to annex 2 of § 2 Abs. 2 Tierschutz-Versuchstier-Verordnung. Animal numbers were reported to the local authority (Regierungspräsidium Darmstadt, approval numbers: V54-19c20/15-F126/1020 and V54-19c20/15-F126/1023).

### Ribo- and RNA-Seq Libraries from Microdissected Rat Somata and Neuropil.

Total Ribo-seq (including monosomes and polysomes) and RNA-seq libraries from microdissected rat somata and neuropil of three biological replicates were generated previously (16) (*SI Appendix*, Table S1). In short, somata and neuropil were microdissected from 4-wk-old male rats. The tissue samples were homogenized in polysome lysis buffer (20 mM Tris pH 7.5, 150 mM NaCl, 5 mM MgCl_2_, 24 U/mL TurboDNase, 100 μg/mL cycloheximide, 1 mM dithiothreitol (DTT), 1% Triton X-100, and protease inhibitor mixture [Roche]) by douncing in a glass homogenizer. After triturating the lysate 10 times using a 23-gauge syringe, samples were chilled on ice for 10 min and cleared by two centrifugations at 16,100 × *g* for 6 min. From the somata and neuropil lysates Ribo-seq and RNA-seq libraries were prepared simultaneously. For Ribo-seq, neuropil and somata lysates containing equal amounts of total RNA were digested with 0.5 U/μg RNase I (Epicentre), shaking for 45 min at 400 rpm at 24 °C. Nuclease digestion reactions were promptly cooled and spun, and 10 μL of SUPERaseIN*RNase inhibitor was added. Samples were then layered onto a 34% sucrose cushion, prepared wt/vol in gradient buffer supplemented with 20 U/μL of SUPERaseIN*RNase inhibitor. 80S particles were pelleted by centrifugation in a SW55Ti rotor for 3 h 30 min at 55,000 rpm at 4 °C. Ribo-seq libraries were prepared according to ref. 61 with the modifications described in ref. 16. Total RNA was isolated from tissue lysates using the Direct-zol RNA micro prep kit (Zymo). RNA integrity was assessed using the Agilent RNA 6000 Nano kit. Rat neuropil and somata total RNA-seq libraries were prepared from an equal amount of total RNA using the TruSeq stranded total RNA library prep gold kit (Illumina) (16). Libraries were sequenced on an Illumina NextSeq500, using a single-end 52- and 75-bp run for Ribo-seq and RNA-seq, respectively.

### RNA-Seq Libraries from Neuron-Enriched and Glia-Enriched Cultures.

Neuron-enriched and glia-enriched cultures were prepared from the same litter as described previously (12). The hippocampi of P0-d-old rat pups were isolated and triturated after digestion with papain. Both cultures were plated on 60-mm cell culture dishes. For the preparation of hippocampal neuron-enriched cultures, cells were plated onto poly-d-lysine-coated 60-mm cell culture dishes and treated as described above with Ara-C (Sigma) at a final concentration of 5 μM for 48 h. After 48 h, the medium was replaced with preconditioned growth medium and cells were cultured until 21 d in vitro (DIV). For the preparation of glia-enriched cultures, cells were plated onto uncoated 60-mm cell culture dishes in conditioned minimal essential medium (minimal essential medium, 10% horse serum, 0.6% glucose [wt/vol]). At 7 DIV, the medium was replaced with preconditioned growth medium and cells were cultured until 21 DIV. Four independent biological replicates were prepared. RNA was isolated using the Direct-zol RNA micro prep kit (Zymo). RNA integrity was assessed using the Agilent RNA 6000 Nano kit. mRNA-seq libraries were prepared starting from ∼200 ng of total RNA, using the TruSeq stranded mRNA library prep kit (Illumina). Libraries were sequenced on an Illumina NextSeq500, using a single-end, 75-bp run.

### RNA-Seq Libraries from Tagged Ribosome Immunoprecipitations.

The input- and translating ribosome affinity purification (TRAP)-seq libraries from hippocampi of Camk2a-Cre-RiboTag or somata/neuropil sections of Wfs1-Cre-RiboTag mice were generated previously (16) (*SI Appendix*, Table S1).

### Ribo-Seq Libraries from Cultured Rat Hippocampal Neurons Treated with Harringtonine.

Dissociated rat hippocampal neurons were prepared from P0-d-old rat pups as described previously (62). Hippocampal neurons were plated at a density of 31,250 cells/cm^2^ onto poly-d-lysine-coated 100-mm dishes and cultured in preconditioned growth medium (Neurobasal-A, B27, GlutaMAX, 30% glia-culture supernatant, 15% cortex-culture supernatant) for 21 DIV. At 1 DIV, cells were treated with Ara-C (Sigma) at a final concentration of 5 μM to prevent the overgrowth of nonneuronal cells. After 48 h, the medium was replaced with preconditioned growth medium and cells were cultured until 21 DIV. Cells were fed with 1 mL of preconditioned medium every 7 d. Three independent biological replicates were prepared. At 24 h before drug treatment, cell medium was adjusted to 8 mL per dish. In appropriate experiments, harringtonine (LKT Laboratories) was added to a final concentration of 2 μg/mL from a 5 mg/mL stock in 100% ethanol. Cells were returned to the incubator at 37 °C for 15, 30, 45, 90, or 150 s. Cycloheximide was added to a final concentration of 100 μg/mL from a stock of 50 mg/mL in 100% ethanol. After drug addition, cells were returned to the incubator at 37 °C for 1 min. After the incubation with cycloheximide, the cells were immediately placed on ice and washed twice with ice-cold phosphate-buffered saline (PBS) plus 100 μg/mL cycloheximide and scraped in polysome lysis buffer (20 mM Tris pH 7.5, 150 mM NaCl, 5 mM MgCl_2_, 24 U/mL TurboDNase, 100 μg/mL cycloheximide, 1 mM DTT, 1% Triton-X-100, and protease inhibitor mixture [Roche]) (21). After scraping, the lysates were triturated 10 times using a 23-gauge syringe; samples were chilled on ice for 10 min and then cleared by centrifugation at 16,100 × *g* for 10 min. Ribo-seq libraries from rat hippocampal neuron cultures treated for 0, 15, 30, 45, 90, and 150 s with harringtonine were prepared as described above. The 0-, 30-, and 90-s datasets were previously published in ref. 16 (*SI Appendix*, Table S1).

### In Situ Hybridization in Hippocampal Brain Slices.

Four-week-old male rats were perfused with 1× RNase-free PBS and fixative solution (4% (vol/vol) paraformaldehyde (PFA), 4% (wt/vol) sucrose in 1× RNase-free PBS). Brains were dissected and fixed for another hour at room temperature. Brains were cryoprotected for two consecutive days at 4 °C. In 15% (wt/vol) sucrose in RNase-free 1× PBS on day 1, followed by 30% (wt/vol) sucrose in RNase-free 1× PBS on day 2. Hippocampi were cryosectioned at 30-μm thickness.

Fluorescence in situ hybridization was performed using the QuantiGene ViewRNA kit (Thermo Fisher) mostly following the manufacturer’s instructions. In brief, hippocampal slices were postfixed for 10 min at room temperature in fixative solution (4% [vol/vol] PFA, 5.4% [wt/vol] glucose, 0.01 M sodium metaperiodate in 1× lysine-phosphate buffer). The manufacturer recommended proteinase K treatment was omitted to preserve the integrity of the dendrites. Slices were permeabilized for 20 min using the kit’s detergent buffer. Detection probes were incubated overnight at 40 °C. Preamplification, amplification, and label probes were incubated for 60 min at 40 °C, respectively, washing three times for 5 min between each step. After completion of in situ hybridization, slices were washed with 1× PBS and incubated in blocking buffer (4% [vol/vol] goat serum 1× PBS) for 1 h at room temperature. The primary antibody (gp-anti-MAP2, SYSY 188004, 1:1,000) was incubated overnight in blocking buffer at 4 °C. Slices were washed five times for 10 min in 1× PBS and the secondary antibody (gt-anti-gp Alexa 647, Thermo Fisher A21450, 1:500) was incubated in blocking buffer for 5 h at room temperature. Slices were washed in 1× PBS and nuclei were stained with DAPI for 3 min at room temperature. Slices were mounted in AquaPolyMount.

Slices were imaged using a Zeiss LSM780 confocal microscope and a 40× oil objective (numerical aperture [NA] 1.3). Z stacks spanning the entire slice volume were obtained using appropriate excitation laser lines and spectral detection windows. The mRNA signal was dilated for better visualization. The raw, nondilated images were used for analysis.

An in-house Python script was used to count mRNA puncta in the somata and the neuropil layer, respectively. In the neuropil, puncta colocalizing with DAPI signal (arising from glia or interneurons) were excluded from the analysis. Counts were normalized by area and a neuropil-to-somata ratio was computed for each slice. The mean neuropil-to-somata ratio was calculated for somata-translation-up target genes. All neuropil-to-somata ratios were divided by this average.

### Data Analysis.

#### Genome and transcriptome alignment of ribosome profiling libraries.

Sequencing adapters were trimmed using the Cutadapt software version 1.15 (63) with the following arguments: —*cut 1–minimum-length 22 –discard-untrimmed –overlap 3 -e 0.2*. An extended unique molecular identifier (UMI) was constructed from the two random nucleotides (nts) of the reverse transcription primer and the five random nucleotides of the linker and added to the FASTQ description line using a custom Perl script. To remove reads originating from noncoding RNA (ncRNA, i.e., rRNA), trimmed reads were aligned to rat ncRNA using Bowtie2 version 2.3.5.1 (–very-sensitive) (64) and aligned reads were discarded. The remaining reads were aligned to the rat genome (rn6) with the split-aware aligner STAR version 2.7.3.a (65) with the following arguments: *–twopassMode Basic –twopass1readsN -1 –seedSearchStartLmax 15 –outSJfilterOverhangMin 15 8 8 8 –outFilterMismatchNoverReadLmax 0.1.* To retrieve transcript coordinates, STAR’s quant mode (–quantMode) was used. Throughout the study, genome alignments were used for differential expression analyses and genomic feature analyses. Transcriptome alignments were used for all other analyses. The STAR genome index was built using annotation downloaded from the University of California Santa Cruz (UCSC) table browser (66). PCR duplicates were suppressed using a custom Perl script and alignments flagged as secondary alignment were discarded before analysis. Only footprints with sizes between 24 and 34 nts were used for analyses.

#### Genome alignment of RNA libraries.

Sequencing adapters and low-quality nucleotides were trimmed using the Cutadapt software version 1.15 (63) with the following arguments: *–minimum-length 25–nextseq-trim = 20*. The trimmed reads were aligned to the rat (rn6) or the mouse (mm10) genome with STAR version 2.7.3a (65).

#### Genomic feature analysis.

The coordinates of genomic features (CDS, 3′ UTR, 5′ UTR, intron) were downloaded from the UCSC table browser in BED format (66). Bedtools version 2.26.0 (67) was used to convert BAM into BED files and to identify reads overlapping with the individual features.

#### Three-nucleotide periodicity.

P-site offsets were defined for different footprint lengths. Each footprint start position defined the footprint frame in reference to the annotated start codon. The footprint reads were virtually back projected over the start codon and the offsets from the start and the end of the read were calculated. We used every read of a given length and accumulated the most probable offset and frame. Next, the P-site position per footprint read was deduced from its length and the previously determined offset. All P-site positions were plotted for 100 nucleotides around the start and stop codons, and the center of a transcript. To correct for differences in translation rates between genes, the P-site coverage of each gene was normalized to its mean footprint coverage. The nucleotide coverage at the 0, 1, and 2 frame positions were assessed. A one-way analysis of variance (ANOVA) was used to determine if the observed frame fraction was different from the expected frame fraction. A significant *P* value rejected the null hypothesis that all frames featured the expected P-site coverage.

#### Genome browser track visualization.

Footprint alignments were converted into the BedGraph file format using Bedtools version 2.26.0 and visualized as custom tracks on the UCSC Genome Browser (68). Footprint coverages were corrected for sequencing depth.

#### Differential expression analysis.

##### RNA-seq and Ribo-seq neuropil:somata ratios.

For both total RNA sequencing and ribosome footprint libraries from the somata and neuropil, the software featureCounts version 2.0.0 (69) was used to calculate counts per gene from reads that were aligned to the rat genome. All annotated transcript isoforms were considered. Raw counts were fed into DESeq2 version 1.30.1 and log fold change (LFC) shrinkage was used (18). Only genes with an adjusted *P* value are displayed in Fig. 1*B*.

##### RiboTag IP:input ratios and neuron-enriched:glia-enriched culture ratios.

The software featureCounts version 2.0.0 (69) was used to calculate counts per gene from reads mapped to the genome (mm10, rn6). All annotated transcript isoforms were considered. Raw counts were fed into DESeq2 version 1.30.1 and LFC shrinkage was used (18).

#### Gene ontology analysis.

Gene ontology analysis was performed for neuropil- and somata-translation-up genes. All detected genes (baseMean greater than zero and with an adjusted *P* value), without the contaminants, were used as background. GO enrichment analysis was performed for the complete cellular component annotation using the PANTHER overrepresentation test (70, 71). The Fisher exact test was used and only GO terms with a false discovery rate (FDR) smaller than 0.05 were considered. The most specific GO terms per branch were retained. The top five GO terms with the highest enrichment scores were visualized.

Gene ontology analysis was performed for uORF-containing transcripts. All detected genes in the neuropil and the somata (baseMean greater than zero), without the contaminants, were used as background. GO enrichment analysis was performed for the complete biological process annotation using the PANTHER overrepresentation test (70, 71). The Fisher exact test was used and only GO terms with an FDR smaller than 0.05 were considered. The most specific GO terms per branch were retained. All significant GO terms were visualized.

Gene ontology analysis was performed for TE_high_ and TE_low_ transcripts. All detected genes in the neuropil and the somata (baseMean greater than zero), without the contaminants, were used as background. GO enrichment analysis was performed for the complete cellular component annotation using the PANTHER overrepresentation test (70, 71). Only GO terms with at least 50 genes in the background set were used in the analysis. The Fisher exact test was used and only GO terms with an FDR smaller than 0.05 were considered. The most specific GO terms per branch were retained. All significant GO terms were visualized.

#### Computation of translational efficiency.

The number of ribosomes per transcript was estimated by integrating Ribo-seq and RNA-seq libraries to calculate TE values in the neuropil. Raw Ribo-seq and RNA-seq counts, falling into gene CDS, were fed into DESeq2 version 1.30.1 and LFC shrinkage was used (18). TE values that were either significantly higher than log_2_(1.5) in the neuropil and the somata or smaller than log_2_(1.5) in the neuropil and the somata were assigned to TE_high_ and TE_low_, respectively [lfcThreshold = log_2_(1.5) with an FDR < 0.05]. Only genes with a baseMean greater than 10 in the neuropil and the somata were considered. An interaction term was added to the experimental design to compare TE values between the neuropil and the somata (72).

#### Translational efficiency of autism genes.

Genes known to be associated with autism spectrum disorders were downloaded from the SFARI Gene database (https://www.sfari.org). Human gene symbols were converted into rat gene symbols. Genes with an SFARI score of 1 and 2 were considered as autism genes.

#### Motif analysis 3′ and 5′ UTR.

RBP motifs (human, rat, and mouse) were downloaded as position weighted matrices from the public ATtRACT database (31). The FIMO tool from the MEME suite version 5.1.1 was used to scan 5′ and 3′ UTRs for motif occurrences, using the default threshold (*P* value = 1e-4) and a precalculated nucleotide background model derived from query sequences (73). Only genes with an RBP motif occurrence were considered for analysis. For each identified RBP motif, the motif-containing genes were grouped and a median TE value was calculated. A Wilcoxon rank sum test was conducted to test if the median TE of a given RBP motif group differed from the median TE of all genes that do not contain the motif.

#### Detection of translated uORFs.

The ORF-RATER pipeline (https://github.com/alexfields/ORF-RATER) was run as previously described (39), starting with the harringtonine 150 s as well as the neuropil and somata BAM files. Note, that it is possible that a translated uORF may be assigned a low score, as ORF-RATER is tuned to indicate the highest-confidence sites of translation, at the expense of an increased false negative rate (74). The following parameters were used: “--codons NTG” for ORF types, “--minrdlen 28 --maxrdlen 34” for harringtonine-treated samples, “--minrdlen 27 --maxrdlen 34” for neuropil and somata samples. Only uORFs with a score of at least 0.7, a length of at least three codons, and at least one count in each of the neuropil and the somata replicates were considered.

#### Relative uORF to CDS ribosome density.

The ribosome density of a uORF or CDS was computed as the number of ribosome footprints divided by the uORF or CDS length, respectively. The relative ribosome density was computed as the uORF ribosome density divided by the CDS ribosome density.

#### Transcript feature analysis.

The 5′ and 3′ UTR lengths were calculated based on the *Rattus norvegicus* annotation version 6 (rn6). The 3′ UTR lengths were corrected in accordance with newly identified 3′ UTR isoforms described in ref. 12. For genes with multiple 5′ UTR isoforms the longest 5′ UTR sequence was chosen, giving priority to curated isoforms. For genes with multiple 3′ UTRs, the most-expressed 3′ UTR isoform was chosen (12).

For the comparison of 5′ UTR lengths between “TE_high_,” “TE_low_,” and “others” only 5′ UTRs with a minimum length of 10 nts and a maximum length of 5,000 nts were considered. For the comparison of 3′ UTR lengths between “TE_high_,” “TE_low_,” and “others,” only 3′ UTRs with a minimum length of 50 nts and a maximum length of 10,000 nts were considered.

For the comparison of 3′ UTR lengths between neuropil-translation-up and somata-translation-up genes, the 3′ UTR isoform with the highest expression in the hippocampus per gene family was considered (12).

#### Metagene analysis and computation of the elongation rate.

The coverage of each gene was projected along the CDS in transcript coordinates (only exons). Genes with CDS lengths shorter than 440 codons were omitted from analysis. Each metagene profile was scaled by the average coverage between codon 400 and 20 codons before the stop codon. For each time point, the metagene profiles were smoothed with a running average window of 30 codons. For each group, the coverage tracks were accumulated, averaged, and normalized to the 0-s condition. A baseline coverage track was defined as 85% of the nontreated sample coverage track. The first positive crossing between the harringtonine-treated coverage track and the baseline coverage track determined the crossing position in codons. Elongation rates were calculated as the slope of a linear regression between the harringtonine incubation times for each track and the crossing position in codons.

#### Statistical analyses.

Statistical significance and the tests performed are indicated in the figure legends. Statistical analysis was performed using MATLAB and R.

## Data Availability

Details about data availability can be found in *SI Appendix*, Table S1. The accession number for the raw sequencing data published previously in ref. 16 is National Center for Biotechnology Information (NCBI) BioProject: PRJNA550323. The accession number for the raw sequencing data reported in this paper is NCBI BioProject: PRJNA634994. All bioinformatic tools used in this study are contained in one modular C++ program called RiboTools. The source code and further notes on the algorithms can be found on our GitHub repository (DOI: 10.5281/zenodo.3579508). Other analysis scripts and codes are available upon request.
